# Facilitators of and obstacles to practitioners’ adoption of harm reduction in cannabis use: a scoping review

**DOI:** 10.1186/s12954-024-01093-9

**Published:** 2024-10-01

**Authors:** Roula Haddad, Christian Dagenais, Jean-Sébastien Fallu, Christophe Huỳnh, Laurence D’Arcy, Aurélie Hot

**Affiliations:** 1 Department of Psychology, Université de Montréal, Montreal, Canada; 2 School of Psychoeducation, Université de Montréal, Montreal, Canada; 3 University Institute on Addictions, Centre intégré universitaire de santé et de services sociaux du Centre-Sud-de-l’Île-de-Montréal, Montreal, Canada; 4 Centre de recherche en santé publique (CReSP), Centre intégré universitaire de santé et de services sociaux du Centre-Sud-de-l’Île-de-Montréal, Montreal, Canada; 5 Department of Psychiatry and Addictology, Université de Montréal, Montreal, Canada

**Keywords:** Harm reduction, Cannabis, Health, Practitioners, Factors, Facilitators, Obstacles, Practice

## Abstract

**Background:**

Cannabis use can generate potential avoidable harms, hence the need for effective preventive measures and treatment. Studies show the efficacy of harm reduction (HR) in minimizing undesirable consequences associated with this use. Despite its proven efficacy, HR in cannabis use remains poorly applied by many health and social services (HSS) practitioners, especially with young people. However, knowledge regarding the underlying reasons for this is limited. To fill this gap, we aimed to identify facilitators of and obstacles to HSS practitioners’ adoption of HR in cannabis use across OECD countries.

**Methods:**

We conducted a scoping review, guided by Arksey and O’Malley’s model. The search strategy, executed on health databases and in the grey literature, captured 1804 studies, of which 35 were retained. Data from these studies were extracted in summary sheets for qualitative and numerical analysis.

**Results:**

Facilitators and obstacles were grouped into four themes: stakeholders’ characteristics (e.g., education, practice experience); clients’ characteristics (e.g., personal, medical); factors related to HR (e.g., perceived efficacy, misconceptions); factors related to the workplace (e.g., type of workplace). Data were also extracted to describe the populations recruited in the selected studies: type of population, clientele, workplace.

**Conclusion:**

Several factors might facilitate or hinder HSS practitioners’ adoption of HR in cannabis use. Taking these into consideration when translating knowledge about HR can improve its acceptability and applicability. Future research and action should focus on this when addressing practitioners’ adoption of HR.

**Supplementary Information:**

The online version contains supplementary material available at 10.1186/s12954-024-01093-9.

## Background

### Cannabis use

Psychoactive substances (e.g., cannabis, alcohol, nicotine, cocaine, heroin) are defined as substances whose use affects mental processes (e.g., perception, cognition, emotions, and mood) and behaviors without necessarily leading to an addiction or a substance use disorder (SUD) [1, 2]. Among these substances, cannabis ranks as the third most consumed psychoactive substance globally among both adults and youth, after tobacco and alcohol [3–5]. Canada presents one of the highest rates of cannabis use among adolescents (15 to 17 years old) and young adults (18 to 24 years old) in the Organisation for Economic Co-operation and Development (OECD) countries [3, 6, 7]. In 2023, 26% of Canadians aged 15 and older reported using cannabis in the past year, up from 22% in 2018, indicating an increase in cannabis use following its legalization [8]. However, these rates might be based on increased ease around revealing cannabis use, as people who experience stigmatization in the healthcare system might be more reluctant to disclose their cannabis use history [9]. In a 2008 study among youth facing psychosocial adjustment difficulties and living in Quebec’s residential treatment facilities, a substantial 78% had already used cannabis, with almost half presenting a problematic use [10]. It is worth noting that our team intend to rely on the results of this study to conduct a future one among health and social services (HSS) practitioners and stakeholders working with youth facing adjustment difficulties in Quebec.

Cannabis use encompasses various methods, including smoking, ingesting, vaporizing, and vaping [3, 6, 7, 11, 12]. Individuals might use non-prescribed cannabis for several purposes, such as pleasure-seeking, coping with difficult situations, self-medicating, curbing the appetite, etc. [13, 14]. Prescribed cannabis use refers to its supervised medical use to treat or improve symptoms associated with a disease or a disorder [14].

Recreational cannabis use does not necessarily lead to a cannabis use disorder (CUD), in that most people use it infrequently with minimal repercussions at lower usage rates [15, 16]. Leung et al. [17] found that, among people who use cannabis, 4 out of 5 will not develop a CUD. Moreover, cannabis is considered to have less severe consequences on an individual and populational level compared to some other substances, such as alcohol and tobacco [18, 19]. Nevertheless, increased cannabis consumption could lead to harmful consequences for health and well-being (e.g., respiratory problems, deterioration of mental well-being, reduced academic performance, unemployment, CUD) [4, 5, 20–24]. CUD is characterized by an ongoing problematic usage pattern generating negative consequences [25]. Given these potential adverse outcomes, it is becoming crucial to establish prevention and intervention programs targeting adults or youth who use cannabis [7, 22].

### Cannabis use reduction and abstinence-based models

In the late 1900s, the United Nations (UN) published three conventions to criminalize the possession, use, and manufacture of illegal drugs: the Single Convention on Narcotic Drugs (1961), the Convention on Psychotropic Substances (1971), and the Convention against Illicit Traffic in Narcotic Drugs and Psychotropic Substances (1988) [26, 27]. These conventions influenced many countries, especially the United States of America, which launched the “War on Drugs” under President Nixon in 1971 [26, 28, 29]. This led to the widespread adoption of abstinence-based models to prevent and address substance use [15, 30]. These models, which focus on complete cessation of substance use, have been foundational in many prevention and intervention programs, including those targeting vulnerable populations such as youth in foster care centers [21]. Despite its potential to reduce the frequency or quantity of substance intake, abstinence-based models for youth have encountered criticism on several fronts [21]. They often fail to equip individuals with skills to manage potential harms, rely on fear-based tactics, do not address pivotal influencers of usage like peer pressure, and show high rates of relapse and dropout [20, 21, 31, 32]. Several studies have shown the limited efficacy of abstinence-based models, especially among youth [33, 34]. For instance, programs like Project DARE (Drug Abuse Resistance Education) and Project ALERT have demonstrated limited short-term effects and sometimes harmful long-term effects, such as lower self-esteem and no significant impact on substance use [33–36]. Moreover, practitioners working with youth in foster care centers face challenges when implementing abstinence-based programs, as these young people find such programs unrealistic and have developed strategies to circumvent their application [13]. Considering the limitations associated with abstinence-oriented programs, it is becoming imperative to implement alternative and more adaptable treatments that are effective for people who use substances, such as harm reduction (HR) [21, 32, 37].

### Harm reduction in cannabis use

#### Description

Harm reduction (HR) in cannabis use aims to minimize the harmful outcomes associated with the substance, spanning the individual, psychological, legal, and social spheres [20, 32, 37]. This model offers a comprehensive public health framework guided by values of pragmatism and humanism, approaching substance use as a long-standing societal reality without moral judgment [5, 15, 31, 38]. Whether for adults or adolescents, HR acknowledges the individual’s autonomy in making choices rather than imposing mandatory cessation, which is particularly beneficial for those who find cessation from substances undesirable or unfeasible [32, 37, 39, 40]. HR in cannabis use also aims to help individuals make informed and responsible decisions to minimize the adverse individual outcomes linked to their usage [15, 37, 38]. While abstinence can be a desirable end goal in HR interventions, it is distinct from HR when it becomes a mandated objective or admission criterion [31].

HR clarifies the concept of safer substance use that is influenced by three interconnected factors, as presented in the “drug, set, and setting” framework: the drug (quantity, frequency of use, tolerance to the substance, combinations with other substances, etc.), the set (e.g., the individual’s physical and mental health status), and the setting (location, time of day, legal regulations, etc.) [5, 41, 42]. For young people, who are often referred to treatment by external sources like parents or schools, HR addresses their ambivalence about discontinuing substance use and considers their personal characteristics (e.g., impulsivity, sensation-seeking), relapse experiences, engagement in treatment, emotional regulation, etc. [23, 40].

#### Effectiveness

Among adults, HR interventions for non-injected drugs have been extensively researched and have shown promising outcomes in diminishing the adverse harms associated with substance use [20, 21, 32]. For adolescents and young adults, the emphasis shifts more toward school-centered initiatives that combine prevention and early interventions strategies [4, 11]. Most studies have tackled alcohol use, with limited attention directed toward cannabis or the implementation and effectiveness of HR-based interventions among youth [4, 11, 20]. Indeed, current school-based HR programs, such as SHAHRP (School Health and Alcohol Harm Reduction Project) in the United Kingdom and SCIDUA (Integrated School- and Community-based Demonstration Intervention Addressing Drug Use among Adolescents) in Canada, have demonstrated effectiveness in fostering safer attitudes toward cannabis or substance use and diminishing adverse outcomes [21, 43, 44]. Consequently, researchers have advocated for integrating HR principles into interventions tailored for adolescents and young adults [13].

#### HR acceptability

Despite its proven effectiveness, HR acceptability remains controversial among HSS practitioners [45, 46]. Our aim in this section is to explore the general acceptability of HR, independently of the substance or the population (i.e., not limited to cannabis). In fact, valuable parallels can be drawn from its acceptability across various substances. This broader scope enriches our understanding and highlights how practitioners’ acceptability of HR is based on its principles rather than the substance involved.

A study conducted by MacCoun [47] confirmed that, among practitioners who did not support HR in substance use, some grounded their choice in moral considerations, regardless of its efficacy [47]. Numerous obstacles constrain its application by healthcare professionals such as the ambiguities in its conceptualization; for instance, some practitioners perceive HR as conveying the wrong message by suggesting a level of tolerance or even endorsement of substance use [31, 32, 48]. MacCoun replied to this critique by suggesting that, if HR service providers wished to convey a message, it might be along the lines of: “[…] if you will not quit using drugs, we can help you to use them less harmfully” (MacCoun, 1998, p. 1202). Moreover, not all professionals perceive complete abstinence from substance use as an objective attainable through HR [49]. Confusion also arises between reducing usage (i.e., frequency and amount) and minimizing harm (i.e., altering consumption practices such as contexts and mixtures to mitigate harmful consequences) [31]. These misconceptions show the necessity for raising awareness, providing training, and offering supervision to practitioners interested in adopting HR [32]. Ethical dilemmas and issues arising from healthcare practitioners’ personal, collective, and professional values and the therapeutic model of abstinence can also impede HR adoption [31]. HR diverges from traditional treatments by permitting risky behaviors and acknowledging HR in drug use as a legitimate outcome [31, 48]. Practitioners may also have concerns regarding potential legal, societal, and health-related problems among their clients [13, 32]. Furthermore, HR adoption must contend with other challenges, including insufficient funding, stigma that undermines demand for care, opposition from local authorities, and lack of services and trained personnel [45].

However, several factors perceived as benefits have been identified as facilitators of HR adoption by healthcare providers. These include a broadening of the range of acceptable objectives, enhancement of clients’ decision-making capabilities, the cultivation of positive and good-quality relationships, and effective management of relapses [32]. A study by Sharp et al. [45] indicated that clarifying the positive impacts of HR (such as safety) at the community level and ensuring the availability of resources could increase the likelihood of its adoption.

#### Purpose of this scoping review

Even though the effectiveness of HR has been established, its implementation faces several limitations, such as HSS practitioners’ reluctance to apply it [31, 45]. To our knowledge, there has been no comprehensive examination of the scientific literature that systematically pinpoints the factors enabling or hindering the adoption of HR in the context of cannabis use. To fill this gap, we aimed to identify, through a scoping review, facilitators of and obstacles to HSS practitioners’ adoption of HR in cannabis use.

## Methods

The detailed research protocol for this study has been published [50]. The study follows the methodological steps of scoping reviews [51, 52]. This type of review has gained prominence in recent times and is categorized among knowledge synthesis reviews [51, 53]. Although there is no universal definition for scoping reviews, several factors set them apart from other types of knowledge syntheses [54]. For instance, scoping reviews tackle broad research questions, encompassing studies with diverse designs and multiple sources of evidence, thereby providing an overarching view of the available literature around a concept [55, 56]. Furthermore, in a scoping review, evaluating the methodological quality of the studies included is suggested but optional [57, 58]. We decided to conduct this type of knowledge synthesis to review research activity in a given area, to summarize and disseminate existing research findings in a subsequent study to practitioners through a knowledge translation process, and to identify literature gaps [55].

The foundational model for conducting scoping reviews was introduced by Arksey and O’Malley [55] and encompasses six stages. Our methodology is guided by this model, which was later refined by Levac et al. [56] and revised by members of the Joanna Briggs Institute (JBI) [59]. The six sequential stages we adhered to are: (1) determining the research question and the objective; (2) identifying relevant studies; (3) selecting studies; (4) charting the data; (5) collating, summarizing, and reporting the results; and (6) conducting a consultation exercise (optional).

The research protocol has been documented following the framework of the Preferred Reporting Items for Systematic Reviews and Meta-Analyses Extension for Scoping Reviews (PRISMA-ScR) grid [53]. This grid serves as an extension of the original PRISMA grid tailored for Cochrane-type systematic reviews and is crucial in upholding the study’s transparency and replicability [53, 58].

## Stage 1: determining the research question and the objective

Initiating a scoping review involves not only delineating the research questions but also clarifying objectives [54, 55, 58]. Our scoping review is fundamentally exploratory, aimed at identifying facilitators of and obstacles to HSS practitioners’ adoption of HR in cannabis use. We drew upon the Population-Concept-Context (PCC) model [58] to formulate the research question: What factors influence practitioners (population) in the health and social services fields (context) to adopt HR in cannabis use (concept)? We also identified specific research questions associated with the components of the PCC model:

- Question 1, related to concept and context: What are the facilitators of and obstacles to HSS practitioners’ adoption of HR in cannabis use?
- Question 2, related to population: Who are the clientele of the HSS practitioners identified in the studies (e.g., adolescents and young adults, pregnant persons, individuals with psychotic disorders, etc.)?
- Question 3, related to concept: What is the definition of HR in cannabis use?

### Stage 2: identifying relevant studies

#### Search strategy

The development of the search strategy followed an iterative approach. Initially, a senior librarian at the Quebec Library on Addictions (*Bibliothèque québécoise sur les dépendances*) crafted three distinct search strategies, each centered around different concepts, which were then tested on the Medline database. The first 50 results from each strategy were assessed, and the strategy was chosen that effectively grouped terms related to concepts of harm reduction, clinicians, and cannabis. The chosen search strategy was then reviewed by a second information professional from the RENARD Research Team on Knowledge Translation, who further adapted and tailored it to align with the designated databases. The Peer Review of Electronic Search Strategies (PRESS) tool was employed by the librarians as a reference during this process [60]. The search strategy, initially executed on Medline and later adapted for other selected databases, is presented in Additional file 1.

#### Information sources

To identify relevant published and unpublished studies, a variety of information sources were explored [51, 55, 58]. With the guidance of the two librarians, the search strategy was executed on October 10, 2022, across prominent health and intervention databases, including Medline, PsycINFO, CINAHL, Web of Science, Embase, and Sociological Abstracts. To explore the grey literature, the search strategy was tailored to suit the Google Web and Google Scholar search engines, along with the Érudit (French database) and BASE databases. It should be noted that several search strategies were developed for the Google Web engine. When these were executed, the results on the first page were consulted, and when these appeared relevant, the following pages were screened until no further relevant results appeared. All identified documents across all databases were organized within Zotero software for convenient access by the research team members. Additional sources of information were also searched to identify any publications overlooked by the electronic searches: after completing the third stage, the reference lists of the selected studies were manually searched, as were the included studies in the identified knowledge synthesis.

### Stage 3: selecting studies

After completing and the second stage, identified duplicates were eliminated. The remaining 1,804 documents were imported into Covidence software. Subsequently, two reviewers (RH, YS) independently read the titles and abstracts of all the identified studies to assess their potential relevance for inclusion based on predefined inclusion and exclusion criteria (Table 1). The two reviewers met regularly to reconcile any discrepancies in the selection process and to adjust the eligibility criteria if necessary. When needed, other reviewers (CD, JSF, CH) were consulted to mediate in the resolution of any conflict. Studies were excluded when they met at least one exclusion criteria. Following this initial phase of screening, the inter-rater agreement between the reviewers was 0.94.

**Table 1:** Inclusion and exclusion criteria

Criteria	Inclusion criteria	Exclusion criteria
Type of study	Empirical study: quantitative, qualitative, or mixed	Study that does not present empirical results (e.g., theoretical study, conceptual framework, etc.) or knowledge review (e.g., systematic or literature review) Interviews conducted outside of an empirical research framework, such as those with journalists
Type of document	Peer-reviewed scientific article, research report, dissertation, thesis	Book, practice guide
Conceptual framework	HR in cannabis use Cannabis risk reduction Non-abstinence in cannabis use	Another conceptual framework
Objective	Identification of factors^1^ facilitating or hindering practitioners’ adoption of the HR approach^2^ in cannabis use	Evaluation of the efficacy of interventions based on HR OR Stakeholder perceptions of the use of cannabis as an HR strategy to circumvent the effects of other drugs OR Attitudes toward decriminalization of cannabis
Psychoactive substance being studied	Marijuana, hashish, or cannabis for non-medical purposes “Drug” if cannabis is part of its conceptualization in the study	Any substance other than marijuana, hashish, or non-medical cannabis (e.g., tobacco, alcohol, medical cannabis, MDMA, Ecstasy) Study that focuses on “performance and image enhancing drugs” or “crack” or “new psychoactive substances”
Target population	Practitioners^3^ working in the health and social fields^4^ Practitioners in training	People who use cannabis^5^
Country of study	OECD country	Non-OECD country
Publication date	From 1990 onwards	Before 1990
Language	French and/or English	Languages other than French or English or text not available

^1^“Factors” include perceptions, beliefs, facilitators, obstacles, oppositions, attitudes, opinions, barriers, biases, motivations, preferences, determinants, incentives, influences, and perspectives on the adoption of HR in cannabis use, as well as its acceptability and receptibility

^2^“Approach” refers to strategies, interventions, practices, services, methods, techniques, treatments, programs, or guides for the HR approach in cannabis use

^3^“Practitioners” includes healthcare and psychosocial services personnel, professionals, or practitioners, allied healthcare personnel, professionals, or practitioners, social workers, counsellors, psychoeducators, educators, nurses, criminologists, psychologists, clinicians, caregivers, therapists, psychotherapists, and physicians

^4^Although health practitioners and social services practitioners are trained in different disciplines, their interdisciplinary collaboration and network-based work can blur the distinctions between their settings. This is why our search strategy included both disciplines, ensuring that we did not omit any relevant studies

^5^Studies addressing the views of people who use cannabis regarding HR or its adoption by practitioners were excluded

**Fig. 1:**
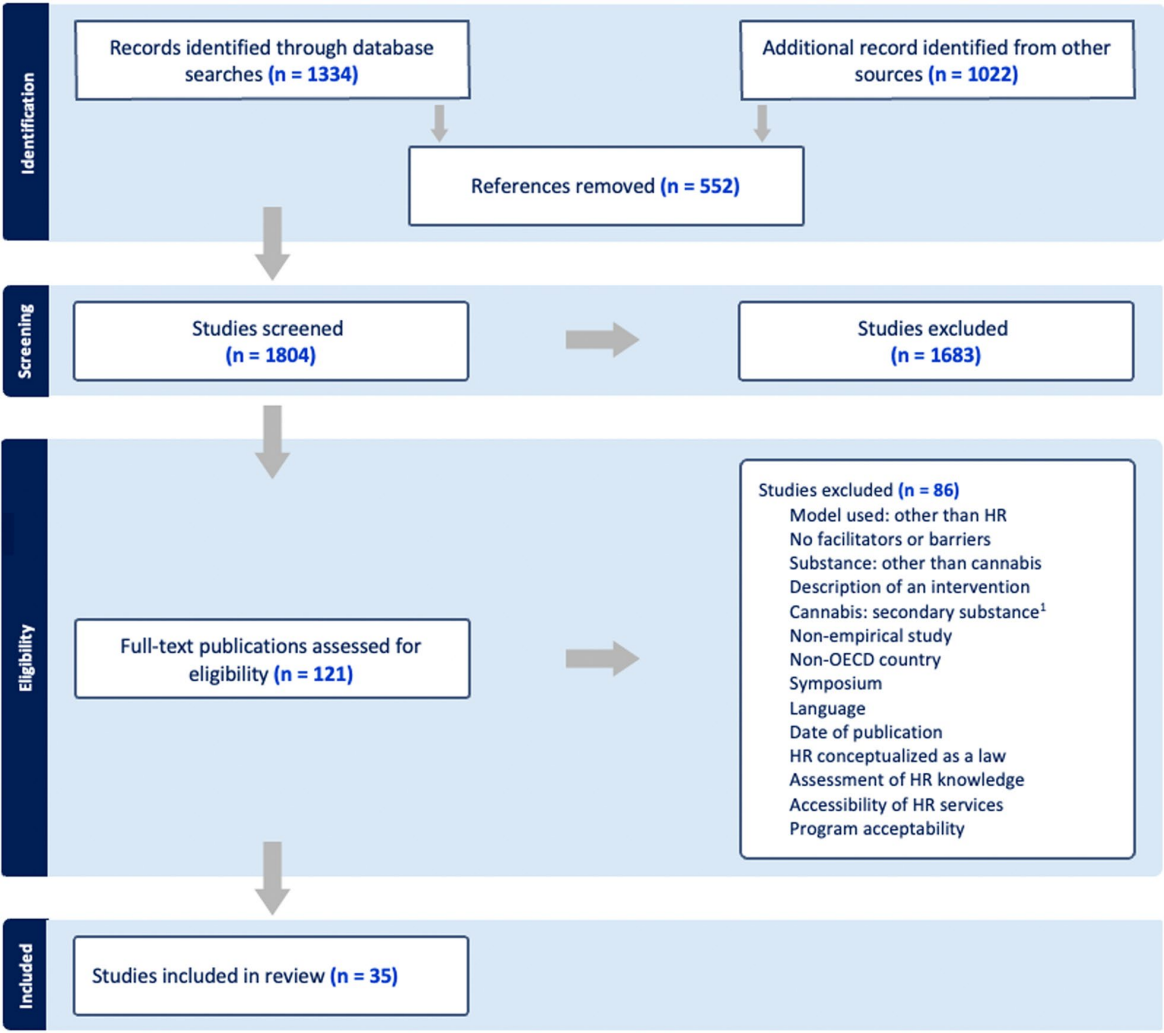
Flow chart detailing identification and selection of studies for inclusion

The 121 documents selected based on potential relevancy were the subject of the next stage, full-text reading. Again, the two reviewers recorded their choices in the Covidence platform, and any new conflicts were addressed in the same way as in the first stage (i.e., titles and abstracts reading). Inter-rater agreement for this step was 0.78, and upon its completion 35 studies were retained for inclusion. The steps in the third stage (i.e., study selection) are represented visually in the following PRISMA diagram (Fig.  1).

#### Inclusion and exclusion criteria

Explicit inclusion and exclusion criteria were defined and adjusted throughout the study selection stage (Table 1). Empirical studies of quantitative, qualitative, or mixed designs, including those published in the grey literature, were selected. Included studies focused on identifying factors that facilitate or hinder HSS practitioners’ adoption of HR in cannabis use^1^. The studies included in our review did not differentiate between CBD and THC products, and thus the review addresses cannabis use in a more generalized manner. To facilitate comparability and extend the applicability of findings to the specific context of Quebec, the review encompassed studies conducted within any of the 38 countries affiliated with the OECD. Articles published from 1990 onwards were included, as that decade (1990s) marked the international emergence of HR and its subsequent expansion. Papers not aligned with these specified inclusion criteria were excluded from consideration. As stated, although systematic reviews were omitted to prevent duplication and ensure equitable representation of the selected papers, their reference lists were scanned to identify potential additional references.

### Stage 4: charting the data

To ensure a consistent analytical approach across the chosen studies, specific variables of interest were determined in accordance with the research questions [55]. These variables were used to generate summary sheets in Microsoft Excel to extract findings (Table 2). This method is an analytical descriptive recording of the data [55, 58, 61]. The primary author (RH) extracted data from the selected studies and created summary sheets. Throughout this process, the research supervisor (CD) validated their accuracy and coherence, and verified their alignment with the original research questions [58].

**Table 2:** Summary sheets

General variables	Specific variables
General characteristics of the study	Study title Author(s) Language of publication Date of publication Period of publication Journal Type of article Full reference Country of study Psychoactive substance under study Legal status of cannabis in country of study
Introduction	Main concepts Definition of the main concept: HR in cannabis use Research question(s) Objective(s) Hypothesis
Methodology	Study design Target population Place of work of the target population Inclusion criteria for participants Recruitment method Sample size Country of origin of participants Clientele of the population recruited Data collection method Analysis steps
Results	Sample presentation Key findings: (1) facilitators and (2) obstacles to practitioners’ adoption of HR in cannabis use Secondary outcomes or other results
Conclusion	Study strengths Study limitations Future research needs and courses of action

### Stage 5: collating, summarizing, and reporting results

Applying the eligibility criteria, selected studies were collected, summarized, and analyzed. To do this, the summary sheets for each selected study were compiled in a table and synthesized (see Additional file 2). This merged table was subject to both a numerical analysis and a narrative organization encompassing a thematic qualitative analysis [52, 53, 55]. The numerical analysis explored the scope, nature, and distribution of the included studies, with a focus on various attributes, such as publication date, country of origin, and document type. Subsequently, RH conducted a thematic analysis, which was then validated by the coauthors (CD, JSF, CH). The analysis involved grouping all findings into themes (e.g., stakeholders’ characteristics, clients’ characteristics), sub-themes, and categories [62]. The objective was to present the data clearly, concisely, and comprehensively, as well as to uncover connections between the collected data and the research questions [62].

### Stage 6: conducting a consultation exercise

Consulting experts is optional, but it enhances the methodological rigor of scoping reviews [55]. For the present study, this step was partially executed; the co-authors (CD, JSF, and/or CH) were invited to serve as consultants to elucidate findings and corroborate the ensuing recommendations. These consultation sessions were held following the acquisition of preliminary results and again upon the conclusion of results analysis.

## Results

In the following section, we will present the general characteristics of the included studies, the facilitators of and obstacles to HR adoption, the stakeholders’ clienteles, and HR definitions. It is important to note that the studies included were conducted among HSS practitioners. However, some studies also recruited other populations (e.g., managers, higher education administrators, etc.). For this reason, when presenting the results, we opted for the term “stakeholders”, which includes HSS practitioners, managers, and any other identified population in the studies. When we specifically refer to “practitioners”, we are focusing exclusively on HSS practitioners and not including other groups, such as managers.

### General characteristics of the studies

Table 3 summarizes the general characteristics of the 35 included studies. Among them, 21 were published between 2011 and 2020, 20 were conducted in the United States, and 30 targeted drugs in general, including cannabis. The studies selected were mainly quantitative (*n =* 16) scientific articles (*n =* 21) published in English (*n =* 32). Cannabis was an illegal substance in 17 studies; its legal status was not mentioned in 11 studies (Table 3).

**Table 3:** General characteristics of the studies (*N =* 35)

	*N =*
**Period of publication** (*N =* 35)	
From 1990 to 2000	1
From 2001 to 2010	7
From 2011 to 2020	21
After 2020	6
**Country of publication** (*N =* 35)	
United States of America	20
Canada	9
United Kingdom	2
United Kingdom and Sweden	1
Australia	1
Ireland	1
Netherlands	1
**Language** (*N =* 35)	
English	32
French	3
**Type of study** (*N =* 35)	
Scientific article	21
Doctoral thesis	8
Master’s thesis	5
Bachelor’s research project	1
**Study design** (*N =* 35)	
Quantitative studies	16
Qualitative studies	14
Mixed studies	4
Randomized controlled trials	1
**Psychoactive substance under study** (*N =* 35)	
Drugs, including cannabis	30
Cannabis	4
Alcohol and cannabis only	1
**Legal status of cannabis in studies** (*N =* 35)	
Not mentioned	11
Illegal	17
Legal	7

### Facilitators and obstacles to HR adoption

The extensive list of factors that facilitate or hinder the adoption of HR is available in Additional file 3.

### Facilitators of HR adoption

#### Theme 1: stakeholders’ characteristics

In relation to theme 1, the facilitators that appeared most to encourage adoption of HR in cannabis use were linked to stakeholders’ level of education: having attended training in SUD or HR (*n =* 5) and holding a high-level degree (Master’s or PhD) (*n =* 3) (Table 4). Other facilitators were weakly identified in the studies, such as having attended conferences and/or courses in HR (*n =* 2), number of years of experience in the substance use field (*n =* 3), and sociodemographic characteristics (e.g., young age of the stakeholder) (*n =* 2). In addition, the ability to apply HR (*n =* 1), stakeholders’ personal characteristics (e.g., being close to a person presenting a SUD) (*n =* 1), as well as their beliefs and perceptions on this topic (e.g., considering that the zero-tolerance approach may have a reverse effect) (*n =* 2), may have contributed to the acceptability of HR in cannabis use. Lastly, eight studies indicated that their recruited populations (practitioners in training, managers, counselors, social workers, and mental health professionals) were open to the idea of adopting HR for cannabis use in their practice, and those studies were conducted in Canada, the United States of America, Australia, and Ireland [32, 63–69].

#### Theme 2: clients’ characteristics

Stakeholders appeared more inclined to adopt HR in cannabis use when clients presented moderate SUDs (*n =* 7) and when HR was used as an intermediate treatment goal (*n =* 6) (Table 4). The client’s psychiatric status (e.g., comorbidity with a psychiatric disorder) (*n =* 2) and personal characteristics (e.g., pregnancy period, young age) (*n =* 2) sometimes favored the acceptability of HR. Other facilitators related to substance use (e.g., presence of non-use days) (*n =* 1) and therapy considerations (e.g., high motivation for change) (*n =* 1) were weakly identified in the studies included.

#### Theme 3: factors related to HR

The facilitators related to theme 3 fall into three categories: (1) HR principles; (2) HR efficacy; and (3) external and other factors.

##### HR principles

The principles that appeared most to promote the acceptability of HR in cannabis use were the ability to focus on clients’ needs and objectives (*n =* 6) and on the present (*n =* 3), and to create a non-judgmental framework (*n =* 4) (Table 4). Other HR principles also appeared to encourage its adoption by stakeholders. HR was perceived as a flexible (*n =* 3), non-punitive (*n =* 3), non-stigmatizing (*n =* 3), preventive (*n =* 3), and motivational intervention or prevention model (*n =* 2). It was opposed to cannabis criminalization (*n =* 2) and valued clients (*n =* 2). In addition, achieving minimal goals was perceived as success, and desired behaviors were rewarded (*n =* 2). Finally, HR was perceived as educating young people about substance use through prevention activities, which enhanced its adoption by professionals (*n =* 4).

##### HR efficacy

The perceived efficacy of HR in cannabis use and its benefits (*n =* 4), especially when abstinence was unattainable (*n =* 2), favored its adoption by stakeholders. Moreover, HR was seen as useful in fostering clients’ engagement (*n =* 8), especially among young people (*n =* 1), as it put them in control of their lives (*n =* 1) (Table 4). Other aspects linked to the HR efficacy in cannabis use made professionals more open to its adoption. For example, HR was seen as an intervention that promotes the therapeutic alliance (*n =* 3), quality of life (*n =* 2), reflection and safe decision-making (*n =* 3), as well as a sense of responsibility and autonomy (*n =* 3). Finally, stakeholders perceived that HR in cannabis use contributed to the reduction and/or control of use (*n =* 4) and minimized symptom severity (*n =* 2). They also considered that HR was aimed at reducing guilt and shame (*n =* 2) as well as harm to the individual (*n =* 2) or to the pregnant person and the fetus (*n =* 3).

##### External and other factors

External factors played a role and appeared to encourage the acceptability of HR in cannabis use, such as the presence of laws that favored its adoption (*n =* 3) and the ineffectiveness of the War on Drugs (*n =* 1) (Table 4). Finally, Moore and Mattaini argued that using a “Consequence Analysis” (CA) instrument made stakeholders more inclined to adopt HR in cannabis use [70]. When asking professionals to respond to a survey on their openness toward HR, the CA method consists of also requesting that they predict each item’s effect (i.e., helpfulness or harmfulness; large or small) on a client; this reflection appeared to generate more openness among professionals toward adopting HR in their practice [70].

#### Theme 4: factors related to the workplace

Management leadership and support were the most cited facilitators related to theme 4 (*n =* 4) (Table 4). In fact, studies revealed that when managers were in favor of HR, gave HSS practitioners the freedom to apply it in their practice, and supported them regularly in its implementation, practitioners felt more at ease and encouraged to adopt it [66, 71]. Working in specific settings (e.g., universities, homeless services, private practice) also appeared to foster HR adoption. In the case of stakeholders undergoing HR training, the qualities of the trainer (e.g., negotiation skills) also sometimes favored its adoption (*n =* 1).

### Obstacles to HR adoption

#### Theme 1: stakeholders’ characteristics

Stakeholders’ lack of training in SUD or HR was the obstacle most frequently cited in the included studies (*n =* 10) (Table 4). In addition, stakeholders with a lower level of education (certificate or bachelor’s degree) appeared more reluctant to adopt HR (*n =* 2). The selected studies also pointed out certain sociodemographic characteristics of stakeholders that might limit HR adoption, such as their living environment (e.g., rural or semi-urban) (*n =* 1) and age (e.g., older age) (*n =* 2). In addition, stakeholders’ beliefs and perceptions (e.g., stigmatizing drug use) (*n =* 2), practice experiences (e.g., lack of skills in applying HR) (*n =* 1), and personal characteristics (e.g., personal history of substance use) (*n =* 1) sometimes also hindered HR acceptability for them. Finally, three studies indicated that their recruited populations were not open to adopting HR and those studies were conducted in the United States of America [72, 73, 92]. These practitioners worked with young people involved in the criminal justice system, pregnant persons, new parents, and/or young adults.

#### Theme 2: clients’ characteristics

Stakeholders appeared less open to adopting HR in cannabis use when clients presented a severe SUD (*n =* 8) and when HR was used as the final treatment goal (*n =* 6) (Table 4). Clients’ personal characteristics (e.g., age between 18 and 30 years) (*n =* 3) and medical (*n =* 2) or psychiatric status (e.g., presence of a comorbidity) (*n =* 3) could also hinder the adoption of HR in cannabis use. Finally, factors related to substance use (e.g., polysubstance use), as well as relational (e.g., relationship status), familial (e.g., family support responsibilities), occupational (e.g., employment status), psychological (e.g., emotional stability status), and social (e.g., size of social network) characteristics were weakly raised in the studies as obstacles.

#### Theme 3: factors related to HR

##### Efficacy of HR and misconceptions

Uncertainties about the efficacy and/or safety of HR (*n =* 6) and misconceptions around HR practices (e.g., lack of knowledge about treatment application) (*n =* 6) were the most frequently cited obstacles related to theme 3 (Table 4). Other misconceptions also limited its adoption, such as the idea that HR conveyed the wrong messages to people who use substances (*n =* 4) or promoted substance use (*n =* 4).

##### External and other factors

Other external obstacles were also cited, such as lack of funding (*n =* 5), lack of research on HR (*n =* 3), and the illegality of cannabis for certain clienteles (*n =* 3) (Table 4). In the case of pregnancy, HR adoption appeared to be limited by the lack of research on its impact on the fetus (*n =* 2) and the ethical dilemmas that could arise (*n =* 2).

#### Theme 4: factors related to the workplace

Workplace philosophies that run counter to HR in cannabis use (*n =* 4) and lack of cooperation and collaboration within the team (*n =* 4) could hinder the adoption of HR (Table 4). Stakeholders in favor of adopting HR in their practice found it difficult to do so when their managers were not aligned with their standpoint or when the workplace position on HR was unclear [32, 66, 74, 75]. Working in certain settings (e.g., detoxification residences, residential rehabilitation services, and community-based organizations) could also limit its adoption. Davis and Rosenberg [74] suggested that fear of losing funding or accreditation if HR was adopted also came into play.

**Table 4:** References for the frequently cited facilitators and obstacles to HR adoption

	Facilitators	Obstacles
*Theme 1: stakeholders’ characteristics*		
Education	Training in SUD or HR	Lack of training in SUD or HR
	[64–66, 70, 76]	[32, 64, 66, 70, 71, 75, 77–80]
	High education level	Low education level
	[64, 67, 73]	[67, 73]
Sociodemographic characteristics	Young stakeholder age	Older stakeholder age
	[73, 74, 81]	
Beliefs and perceptions	Considering that the zero-tolerance approach may have a reverse effect	Maintaining personal beliefs stigmatizing drug use
	[63, 82]	[75, 81]
*Theme 2: clients’ characteristics*		
Factors related to SUD	SUD severity: moderate	SUD severity: severe
	[74, 76, 81, 83–86]	[74, 76, 81, 83–87]
Therapy considerations	HR used as intermediate treatment goal	HR used as final treatment goal
	[74, 76, 81, 83–86]	[74, 76, 81, 83, 84, 86]
Medical and/or psychiatric status	Comorbidity with a psychiatric disorder	
	[32, 64]	[71, 74, 75, 84]
*Theme 3: factors related to HR*		
HR principles	HR focuses on clients’ needs and objectives	
	[32, 66, 69, 76, 79, 83, 87, 89]	
	HR creates a nonjudgmental framework	
	[32, 77, 80, 89]	
HR efficacy and/or misconceptions	HR efficacy	Misconceptions related to HR
	Perceptions of HR benefits and efficacy	Uncertainties about HR efficacy and/or dangerousness
	[32, 64, 72, 91]	[32, 64, 74, 76, 78, 80]
	HR seen as fostering clients’ engagement	Misunderstanding HR practices (e.g., lack of knowledge about treatment application)
	[32, 64, 66, 75, 79, 88, 90, 91]	[32, 64, 71, 78, 79, 89]
External and other factors	Laws that favor HR adoption	Illegality of cannabis among a specific clientele
	[66, 69, 82]	[74, 79, 82]
*Theme 4: factors related to the workplace*		
General factors	Management leadership and support	Workplace philosophies that run counter to HR
	[66, 71, 85, 91]	[32, 71, 74, 88]

### Stakeholders’ clienteles

The second research sub-question sought to investigate the “population” component of the PCC model: “Who are the clientele of the HSS practitioners identified in the studies?” To provide a complete and detailed response to our sub-question, we decided to tackle three variables of interest rather than one: (1) the HSS practitioners’ clienteles; (2) the studies’ populations (i.e., practitioners, stakeholders); and (3) the workplace (see Additional file 4).

First, the populations recruited in the selected studies worked mainly with adults (*n =* 10), young adults (*n =* 9), and adolescents (*n =* 6). Some practitioners worked with other specific clienteles, such as individuals with a mental health disorder or from communities of color, pregnant persons, young people involved in the criminal justice system, young adults with a first psychotic episode, polysubstance users, HIV-positive persons, new parents, homeless persons, and prison or probationary populations (see Additional file 4).

Second, the selected studies were carried out with professionals occupying a variety of functions, and some targeted more than one population. Mental health professionals (e.g., therapists, psychotherapists, psychologists, clinicians, addiction specialists) (*n =* 16), counselors (*n =* 10), and managers (*n =* 8) were the most frequently recruited stakeholders. Other populations were also targeted in some of the selected studies, such as social workers (*n =* 8), front-line healthcare workers (e.g., nurses) (*n =* 8), university students (i.e., practitioners in training) (*n =* 3), psycho-educators or educators (*n =* 1), police officers (*n =* 1), health professionals (e.g., physicians, psychiatrists) (*n =* 1), school staff (e.g., principals, teachers) (*n =* 1), and higher education administrators (e.g., deans, directors) (*n =* 1) (see Additional file 4).

Third, the workplaces in which the recruited stakeholders worked most were: outpatient agencies for SUDs and/or mental health disorders (*n =* 10); inpatient or residential addiction rehabilitation services (*n =* 9); community-based organizations (*n =* 8); and private practices (*n =* 8). Workplaces mentioned moderately were: detoxification residences; halfway houses; hospitals; universities; and prison settings. The least-cited settings were: schools; inpatient agencies (public and/or private) for SUDs and/or mental health disorders; homeless services; assessment, referral, and counseling services; the criminal justice system; early intervention services for first-episode psychosis; public agencies; agencies for pregnant persons with SUDs; and the police sector (see Additional file 4).

### HR definitions

Given the lack of consensus on the definition of HR, our review aimed to address this issue and examine how it is conceptualized across different studies. This also enabled us to identify and compare the definitions of HR used in the included studies, ensuring a clearer understanding of how the concept is conceptualized across various studies.

To answer the third research sub-question related to the “concept” component of the PCC model (“What is the definition of HR in cannabis use?”), we first collected the definitions cited in the studies and then analyzed them in a descriptive qualitative manner. By coding the definitions, we were able to identify recurring commonalities. However, 12 studies did not define HR, focusing instead on its acceptability to stakeholders or people who used psychoactive substances. In the studies that did define HR (*n =* 23), the focus was either on: (1) HR conceptualization; (2) HR principles; or (3) HR efficacy (see Additional file 5).

First, some studies pointed out that it is often designated as a “non-abstinence model” (*n =* 2) and does not have a universal definition (*n =* 1). Second, the studies that defined it by relying on HR principles mainly emphasized the notions that HR does not primarily focus on substance abstinence (*n =* 5), that it originates from the field of public health (*n =* 5), and that it broadens the spectrum of intervention goals deemed acceptable in HR treatments (*n =* 4). Furthermore, HR enables access to health services (*n =* 3) and implements educational and preventive strategies (*n =* 4) that promote a sense of control or self-efficacy regarding the initiation and/or cessation of use (*n =* 3). HR has also been defined as a pragmatic (*n =* 2) and non-stigmatizing model (*n =* 2) (see Additional file 5). Third, some studies based their definitions of HR on its efficacy. For example, HR has shown potential for reducing the negative legal, medical, professional, social, economic, and/or family harms of the substance (*n =* 15). It is a model that enables moderate or controlled substance use (reduced amount and/or less frequent use) (*n =* 6) and that ensures safe, secure, and enjoyable use (*n =* 6) (see Additional file 5).

## Discussion

HR is a prevention and intervention model aimed at helping individuals moderate and control their substance use while applying safety measures to reduce the harms of the substance on several levels (e.g., legal, medical, professional, social, economic, relational) [84, 92]. The main objective of this scoping review was to identify facilitators of and obstacles to HSS practitioners’ adoption of HR in cannabis use in OECD countries. Several factors related to stakeholders’ and clients’ characteristics, to HR attributes, and to the workplace, were found to play a role. To explore our sub-research questions, we retrieved HR definitions as articulated by the authors of the included studies and presented the populations recruited in each study as well as their clienteles and workplaces.

### Stakeholders’ educational background

Stakeholders’ educational background appeared to play an important role in their adoption of HR in cannabis use. Having attended a training program in SUD or HR and holding a high-level degree (master’s or PhD) facilitated HR adoption. In contrast, stakeholders who lacked training in this domain and who held lower levels of education (certificate or bachelor’s degree) were more likely to be opposed to applying HR in cannabis use in their practice. These findings support certain courses of action identified by other researchers, who point out the need to overcome the lack of knowledge about HR in cannabis use by organizing training, for example, or by clarifying the guidelines for safe cannabis use [32, 64, 66, 75, 78, 79, 81, 89, 92]. A systematic review on education for HSS practitioners revealed that those who pursued higher education not only nourished their critical thinking ability, but also tended to be more open to questioning the effectiveness of their previous practice and modifying it accordingly, if necessary [93]. This finding confirms our results and can serve to better understand them. In other words, practitioners completing an advanced level of education might find themselves reflecting on the efficacy of their previous practice founded on the abstinence-based model and becoming more open to learning and applying new evidence-based practices (i.e., HR).

However, achieving a higher level of education may not be feasible for several reasons (e.g., time, cost) for many HSS practitioners. To address this reality, institutions and organizations could consider several strategies to make HR training more accessible to practitioners with lower education levels. For instance, they can offer additional training opportunities by creating concise, accessible training modules, offering mentorship programs, and orienting individuals to available training opportunities. Developing alternative training formats that cater to diverse learning needs can also ensure that those less inclined towards HR practices receive the necessary support.

### Stakeholders’ clienteles

It is worth noting that our study does not aim to identify the specific factors that facilitate or limit the adoption of HR in cannabis use based on the type of clientele. Instead, it provides a general overview. Some of the included studies mentioned facilitators and obstacles related to different client populations, and these will be discussed in the present section. However, not all of the included studies provided detailed information on these aspects.

Our findings reveal that working with certain clienteles affect the acceptability of HR in cannabis use by stakeholders. For example, HSS practitioners are encouraged to adopt HR in cannabis use while working with pregnant persons because it reduces the harms of the substance on both the pregnant person and the fetus, lessens the sense of shame and guilt, and enhances the client’s engagement in the treatment. Conversely, some practitioners might be reluctant to apply HR in cannabis use with this clientele due to lack of training and poor comprehension of HR practices during pregnancy, the lack of research on the impact of HR during this period, and the ethical dilemmas that might emerge. In fact, facing ethical dilemmas when applying HR and assessing the potential risks that might emerge are among the influential factors that might limit its implementation with a pregnant person [31, 32, 94].

When working with adolescents or young adults between 18 and 24 years of age, some stakeholders tended to accept HR in cannabis use because it is non-stigmatizing, focuses on the youth’s objectives and needs, and thereby enhances their commitment to the treatment. On the other hand, some stakeholders were opposed to applying HR in cannabis use with this young population due to the illegality of cannabis in their regard, concerns about sending the wrong messages, lack of training and poor understanding of HR techniques, or the presence of a non-HR workplace philosophy. These findings are aligned with future research needs as formulated by other researchers on this topic, who stress the need to determine whether practitioners’ attitudes towards HR are in line with their work and/or academic environment, the legal status of the substance, and other potentially influential factors [63, 73, 82–85, 89].

Understanding the factors that facilitate and limit the adoption of HR in cannabis use among youth can provide valuable insights to optimize its applicability. For instance, addressing concerns about the legality of cannabis and clarifying HR principles can help mitigate some of the obstacles. Additionally, providing targeted training and resources to practitioners can enhance their ability to effectively apply HR. Practitioners can also use these insights to reflect on their own practices, identify potential obstacles to the adoption of HR among certain groups, and work to enhance its applicability. Future actions should be informed by these empirical findings to develop strategies that address the unique challenges and leverage the opportunities for HR adoption among youth.

### Severity of SUDs and other comorbidities

In this scoping review, HR in cannabis use adoption appeared to be facilitated among practitioners working with clients presenting a moderate SUD (i.e., not severe) and when HR was used as an intermediate, rather than final, treatment goal. This result applied to those working with any clientele and corroborated the findings in our literature review cited earlier. In fact, the long-standing War on Drugs policies and the conceptualization of addiction as a disease sometimes made it harder for practitioners to perceive HR as a legitimate treatment goal [31, 32]. A national study excluded from this scoping review because it was conducted in a non-OECD country (Ukraine) also found that addiction treatment providers were more prone to apply HR in the case of harmful cannabis use than for cases of dependence [95]. However, in this Ukrainian study, practitioners were more inclined to adopt HR as a final, rather than intermediate, treatment goal when working with clients presenting harmful cannabis use, which is contradictory to our findings [95].

Furthermore, we were not able to conclude on the impact of clients’ psychiatric conditions on stakeholders’ adoption of HR in cannabis use. Some of the included studies found that practitioners were more encouraged to apply HR with clients who presented a psychiatric comorbidity, whereas other studies indicated that this factor acted as an obstacle to HR acceptability. However, in the presence of a psychiatric comorbidity, we noted that the practitioners inclined toward adopting HR in cannabis use were those working in organizations serving people who use substances (e.g., community agencies or services for homeless populations), whereas those opposed to it were employed in governmental institutions (e.g., outpatient agency for SUD and/or mental health, hospital, halfway house, prison, detoxification residence, inpatient or residential addiction rehabilitation services, private practice). It should also be noted that, while the presence of a psychiatric condition might facilitate or hinder HR adoption, the presence of a medical condition was only considered an obstacle.

### Impact of perceived HR in cannabis use efficacy

As stated by Lauritsen [85], it is important not to confuse HR “acceptability” and “perceived efficacy”; these are two different concepts, although one can affect the other. Stakeholders who perceived the benefits of HR in cannabis use, in terms of its capacity to focus on clients’ needs and objectives in the present moment while creating a non-judgmental environment, tended to be more accepting of it. In contrast, practitioners were reluctant to adopt HR in cannabis use when they were uncertain about its efficacy or did not understand its practices. HSS practitioners sometimes perceived HR as ambiguous and requiring tangible implementation methods, which could hinder its acceptability [32]. This being said, poor understanding of HR applicability could continue to limit its use by practitioners and lead them to adopt models that are clearer but with limited efficacy, such as abstinence-based models.

Based on these findings, several actions can be taken to increase HR acceptability and perceived efficacy, ultimately leading to more effective treatment outcomes. Organizations might benefit from (1) offering or directing practitioners to HR training programs and ensuring that they are knowledgeable about HR, its applicability, and effectiveness; (2) defining and communicating their stance on the adoption of HR to implement consistent practices; (3) designating a resource person who can answer practitioners’ questions and provide clinical support; and (4) facilitating discussions about beliefs and perceptions related to HR. Ensuring a shared and accurate understanding of the approach among all practitioners can improve its implementation and acceptability.

When discussing the impact of perceived HR efficacy, it is important to keep in mind the concept of Consequence Analysis (CA) raised in one of the included studies [70]. As mentioned earlier, integrating the CA method into an HR questionnaire consists of asking participants not only to respond to the questions, but also to predict each item’s effect (i.e., helpful or harmful; large or small) on the client [70]. When doing this, practitioners are led to step back, reflect on the effect of each statement, and perceive the benefits and usefulness of HR, so they can become more open toward it [70]. This outcome led Moore and Mattaini [70] to recommend that researchers apply the CA method in future studies quantitively assessing HR acceptability among practitioners and/or stakeholders.

### Impact of external factors on HR in cannabis use adoption

External factors were also found to affect HR in cannabis use acceptability, such as laws regarding its adoption, the legal status of cannabis in relation to certain clienteles, the funding situation, or the availability of research on HR. However, we were not able to conclude on the impact of cannabis legalization on attitudes toward HR. Some studies found that practitioners were more persuaded to apply HR when cannabis was legal, whereas in others, practitioners expressed acceptance of HR even when cannabis was illegal. While our study does not definitively determine if cannabis legalization facilitates HR adoption, the legal context in countries like Canada offers a valuable opportunity to enhance HR initiatives. Legalization can create a supportive environment for HR by fostering open dialogue about cannabis use, increasing public awareness, and facilitating funding and resources for HR programs. This framework can also help tailor HR strategies to specific regional needs. This validates the need to further study the impact of cannabis legalization on HR implementation [85, 89].

Moreover, working in a place that does not clearly favor the adoption of HR in cannabis use, might limit its applicability by practitioners. Practitioners are more encouraged to apply HR in their practice when their organization supports it. For example, a study conducted among practitioners working with youth in a residential treatment facility in Quebec recommended adopting a comprehensive HR policy and developing a common vision of their mandate; upon doing this, practitioners became for open to applying HR [46]. As stated earlier, some HSS practitioners had concerns about ending the treatment upon achieving HR goals because they conceptualized it as opposed to the traditional therapeutic model of abstinence; this underscores the need for institutional approval to apply HR [31, 32].

### Strengths and limitations

This study encompasses several strengths, as we rigorously followed the six stages conceptualized by Arksey and O’Malley [55] for conducting scoping reviews.

First, we formulated the research question (i.e., stage 1) on the basis of the PCC model, as it is more suitable for scoping studies than the PICO (Patient-Intervention-Comparison-Outcome) model. The PCC model allows the broad scope of the study to be respected without specifying restrictive inclusion criteria, as are required in Cochrane-type systematic reviews [58]. Specific research questions associated with the components of the PCC model were also formulated to further deepen our findings.

Second, we undertook a thorough process to identify relevant studies for inclusion (i.e., stage 2), which allowed us to retrieve a prominent number of results. Using the PRESS tool, two information specialists contributed to the development of the search strategy, which was adapted to several health databases [60]. Additional studies were also identified by searching the grey literature and consulting the reference lists of the included studies.

Third, two reviewers worked independently throughout the entire study selection stage (i.e., stage 3). The inter-rater agreement between them was high, showing the clarity and comprehensibility of the inclusion and exclusion criteria specified. Despite the presence of specific eligibility criteria, the number of included studies (*N =* 35) is high, reflecting the richness of the results and their potential to contribute to the advancement of scientific knowledge. Another strength is the framework that includes all the facilitators of and obstacles to HR adoption, independently of their type or the population or clientele found in the identified studies.

Fourth, we extracted from the included studies not only facilitators of and obstacles to HR adoption, but also the various definitions of HR and the characteristics of the populations studied (i.e., stage 4).

Finally, after the data had been extracted and merged, we conducted both a numerical quantitative analysis and a thematic qualitative analysis to ensure the rigor of the analysis process (i.e., stage 5). The quantitative analysis provided an overview of the general characteristics of the selected studies, while the thematic qualitative analysis facilitated the grouping of results into themes and sub-themes, presenting them in a summarized, clear, and coherent manner (Table 4) (see Additional file 3).

However, this scoping study presents some limitations. Steps could have been implemented to identify additional studies, such as not limiting the publication language to English or French. Moreover, despite the relevance of including only studies conducted in OECD countries to be able to generalize the results to Canada’s reality, omitting this inclusion criterion would potentially have provided additional results. Contacting authors or organizations working in the HR field might also have led us to include other unpublished studies. Furthermore, even though assessing the methodological quality of the included studies remains an optional step in scoping studies aiming to map the current literature on a specific subject, it would have been preferable to undertake it using the Mixed Methods Appraisal Tool [96]. Finally, some of the selected studies tackled drugs in general (including cannabis) and thus did not clearly distinguish between facilitators of and obstacles to cannabis-specific or other drug-related HR practices.

## Conclusion

Cannabis use remains highly prevalent among adults and youth and can generate potential harms to the individual on several levels [3–5, 7, 22]. Due to the limited efficacy of abstinence-oriented programs usually implemented among people who use substances, encouraging HSS practitioners’ adoption of HR remains essential [21, 32, 37]. For this, an understanding of the factors that facilitate or hinder HR acceptability is vital. In our extensive literature search, we found several factors that affect HR acceptability, related to stakeholders’ characteristics, clients’ characteristics, HR attributes, and/or the workplace. Stakeholders’ educational backgrounds, their clienteles, the severity of clients’ SUDs, as well as the purpose of applying HR in treatment should be taken into consideration when assessing facilitators of and obstacles to the adoption of HR in cannabis use. Perceiving the benefits of adopting HR, having laws that support its use, and working in a place that encourages it also shape stakeholders’ attitudes toward HR. However, lack of knowledge about HR hinders its acceptability and, by extension, its adoption.

We consider that some courses of action should be taken firmly into consideration in future research, such as training HSS practitioners to apply HR and clarifying its practices. In addition, the misconceptions associated with HR principles, practices, and efficacy lead us to highlight the need for a knowledge translation process aimed at HSS practitioners and stakeholders. This would help to clarify HR guidelines and applicability, which could improve HR adoption by practitioners.

The inclusion of studies conducted in OECD countries will allow the results to be generalized to the 38 OECD countries and hence, to the reality of Canada and Quebec. However, even with this inclusion criterion, the results could still be useful and potentially generalizable elsewhere. This scoping review will help researchers better address the adoption of HR in cannabis use. Clarification of the facilitators of and obstacles to the adoption of HR in cannabis use can help knowledge translation specialists tackle HR applicability among practitioners and stakeholders more effectively. This could enhance HR implementation, especially in the presence of evidence-based data that show the effectiveness of adopting HR with people who use substances.

## Supplementary Information

Additional file 1

Additional file 2

Additional file 3

Additional file 4

Additional file 5

## Data Availability

All data generated or analyzed during this study are included in this published article (and its supplementary information files). Additional search strategies executed on the databases are available from the corresponding author on reasonable request.

## Abbreviations

CUD: Cannabis use disorder
HR: Harm reduction
HSS: Health and social services
OECD: Organisation for Economic Co-operation and Development
PCC: Population-concept-context
PICO: Population-intervention-comparison-outcome
PRESS: Peer review of electronic search strategies
PRISMA-ScR: Preferred reporting items for systematic reviews and meta-analyses extension for scoping reviews
SCIDUA: Integrated school- and community-based demonstration intervention addressing drug use among adolescents
SHAHRP: School Health and Alcohol Harm Reduction Project
SUD: Substance use disorders

## Footnotes

1 We excluded studies where cannabis was used as a harm reduction strategy to mitigate the harms of other substances. We focused instead on studies that addressed harm reduction directly related to cannabis use itself.
