# Supplementary material for: Facilitators of and obstacles to practitioners’ adoption of harm reduction in cannabis use: a scoping review
Source: Harm Reduct J. 2024 Oct 1;21:178. doi: 10.1186/s12954-024-01093-9 (PMC11445962; doi:10.1186/s12954-024-01093-9)
Supplement: Supplementary file 1 — Additional file 1 [file 12954_2024_1093_MOESM1_ESM.docx]

**Facilitators of and obstacles to practitioners’ adoption of harm reduction in cannabis use: a scoping review**

Roula Haddad, Christian Dagenais, Jean-Sébastien Fallu, Christophe Huỳnh, Laurence D’Arcy, Aurélie Hot

Correspondence to Roula Haddad; [roula.haddad@umontreal.ca](mailto:roula.haddad@umontreal.ca)

**Additional file 1: Medline Search Strategy**

Searched online: 10/10/2022

Number of results: 251

| # | Concept | Equations | Results |
| --- | --- | --- | --- |
| 1 | Harm reduction | Harm Reduction/ | 3898 |
| 2 |  | Risk Reduction Behavior/ | 14132 |
| 3 |  | (protective adj2 strateg*).ab,kf,ti. | 2818 |
| 4 |  | ((reduc* or minimi*) adj5 (harm? or harmful or risk?)).ab,kf,ti. | 239459 |
| 5 |  | 1 or 2 or 3 or 4 | 251821 |
| 6 | Clinicians | exp Health Personnel/ | 588634 |
| 7 |  | Social Workers/ | 971 |
| 8 |  | Counselors/ | 541 |
| 9 |  | exp Health Occupations/ | 1819039 |
| 10 |  | exp Allied Health Occupations/ | 52626 |
| 11 |  | exp Allied Health Personnel/ | 53169 |
| 12 |  | (worker? or psychoeducator? or psycho-educator? or educator? or nurse? or criminologist? or psychologist? or clinician? or practitioner? or physician? or professional? or provider? or co?nselor or co?nselors or caregiver? or giver? or therapist? or psychotherapist? or staff? or personnel? or employee? or doctor?).ab,kf,ti. | 2002544 |
| 13 |  | 6 or 7 or 8 or 9 or 10 or 11 or 12 | 3625104 |
| 14 | Cannabis | Cannabis/ | 12307 |
| 15 |  | "Marijuana Use"/ | 1689 |
| 16 |  | Marijuana Abuse/ | 6905 |
| 17 |  | Marijuana Smoking/ | 5425 |
| 18 |  | (mari?uana or cannabis or hashish or Pot or weed or tetrahydrocannabinol or THC or CDB or cannabidiol or cannabinoid?).ab,kf,ti. | 100005 |
| 19 |  | 14 or 15 or 16 or 17 or 18 | 102988 |
| 20 | Strategies | ("strateg*" or "approach*" or intervention? or prevent* or practice? or service? or "method*" or technique? or tactic? or co?nseling or treatment? or program? or "guide*").ab,kf,ti. | 15074452 |
| 21 | Combination of 3 concepts | 5 and 13 and 19 | 272 |
| 22 | Limit date | limit 21 to yr="1990 -Current" | 272 |
| 23 | Limit language | limit 22 to (english or french) | 263 |
| 24 | Filter OECD |  |  |
| 25 | TOTAL | 23 not 24 | 249 |
|  |  |  |  |
| 22 | Combination of 4 concepts | 5 and 13 and 19 and 20 | 251 |
