# Supplementary material for: Facilitators of and obstacles to practitioners’ adoption of harm reduction in cannabis use: a scoping review
Source: Harm Reduct J. 2024 Oct 1;21:178. doi: 10.1186/s12954-024-01093-9 (PMC11445962; doi:10.1186/s12954-024-01093-9)
Supplement: Supplementary file 2 — Additional file 2 [file 12954_2024_1093_MOESM2_ESM.docx]

**Facilitators of and obstacles to practitioners’ adoption of harm reduction in cannabis use: a scoping review**

Roula Haddad, Christian Dagenais, Jean-Sébastien Fallu, Christophe Huỳnh, Laurence D’Arcy, Aurélie Hot

Correspondence to Roula Haddad; [roula.haddad@umontreal.ca](mailto:roula.haddad@umontreal.ca)

**Additional file 2: Narrative organization of the included studies**

| **Data** | **Study 1** | **Study 2** | **Study …** |
| --- | --- | --- | --- |
| Type of publication |  |  |  |
| Date of publication |  |  |  |
| Country of study |  |  |  |
| Legal status of cannabis in the country of the study |  |  |  |
| Definition of HR in cannabis use |  |  |  |
| Design of the study |  |  |  |
| Target population |  |  |  |
| Place of work of the target population |  |  |  |
| Clientele of the target population |  |  |  |
| Data collection method |  |  |  |
| Key findings: |  |  |  |
| - Facilitators or enabling conditions |  |  |  |
| - Obstacles or adverse conditions |  |  |  |
| Secondary outcomes |  |  |  |
