# Supplementary material for: Facilitators of and obstacles to practitioners’ adoption of harm reduction in cannabis use: a scoping review
Source: Harm Reduct J. 2024 Oct 1;21:178. doi: 10.1186/s12954-024-01093-9 (PMC11445962; doi:10.1186/s12954-024-01093-9)
Supplement: Supplementary file 3 — Additional file 3 [file 12954_2024_1093_MOESM3_ESM.docx]

| **Facilitators of and obstacles to practitioners’ adoption of harm reduction in cannabis use: a scoping review**  Roula Haddad, Christian Dagenais, Jean-Sébastien Fallu, Christophe Huỳnh, Laurence D’Arcy, Aurélie Hot    Correspondence to Roula Haddad; [roula.haddad@umontreal.ca](mailto:roula.haddad@umontreal.ca)  **Additional file 3: Full list of facilitators and obstacles to the adoption of HR in cannabis use** | | |
| --- | --- | --- |
|  | **Facilitators** | **Obstacles** |
| **Theme 1 : Stakeholders’ characteristics** | | |
| Education | Training in substance use disorders (SUD) or harm reduction (HR) | Lack of training in SUD or HR |
|  | (Clark & Wyllie, 2014; Housenbold Seiger, 2005; Kapur, 2016; Moore & Mattaini, 2014; Rosenberg & Davis, 2014) | (Barbosa-Leiker et al., 2022; Broussard, 2019; Clark & Wyllie, 2014; Kapur, 2016; Long, 2016; Mancini et al., 2008; Moore & Mattaini, 2014; O’Leary et al., 2018; Oluwoye & Fraser, 2021; Sauvageau, 2018) |
|  | High level of education (Master’s or PhD) | Low level of education (certificate or bachelor’s degree) |
|  | (Clark & Wyllie, 2014; King, 2020; Richards et al., 2021) | (King, 2020; Richards et al., 2021) |
|  | Having attended conferences and/or courses in HR |  |
|  | (Clark & Wyllie, 2014; Kapur, 2016) |  |
|  | Certification in SUD |  |
|  | (Moore & Mattaini, 2014) |  |
|  | Field of study: Law, addiction, psychology, social work | Field of study: Nursing, medicine, education |
|  | (Abbott-Chapman et al., 2007; Davis & Lauritsen, 2016) |  |
| Living environment | Urban | Rural or semi-urban |
|  | (Kyser, 2010) | |
| Practical experiences | Ability to apply HR | Lack of ability to apply HR |
|  | (Oluwoye & Fraser, 2021) | |
|  | High number of years of experience in the SUD field | High workload with SUD cases |
|  | (King, 2020; Kyser, 2010; Moore & Mattaini, 2014) | (Vayda, 2016) |
|  |  | Likelihood of working with adolescents in future career |
|  |  | (Abbott-Chapman et al., 2007) |
|  |  | Working using the recovery model |
|  |  | (Waterhouse, 2020) |
| Socio-demographic characteristics | Young age of the practitioner | Advanced age of the practitioner |
|  | (Davis & Rosenberg, 2013; Richards et al., 2021; Xin et al., 2022) | |
| Personal characteristics | Being close to a person presenting an SUD | Personal history of substance use |
|  | (Kyser, 2010) | (Rosenberg & Davis, 2014) |
|  | Humility, compassion, flexibility, patience, respect, and hope for others | Low staff morale |
|  | (Kapur, 2016) | (O’Leary et al., 2018) |
| Beliefs and perceptions | Considering that the zero-tolerance approach may have a reverse effect | Stigmatizing drug use |
|  | (Abbott-Chapman et al., 2007; Waterhouse, 2020) | (O’Leary et al., 2018; Xin et al., 2022) |
|  | Acceptability of other HR interventions (behavioral, pharmaceutical, etc.) | Beliefs about the causes of addiction |
|  | (Lauritsen, 2017; Ogborne & Birchmore-Timney, 1998) | (Kapur, 2016) |
|  | Perception of substance abuse as a social and mental health issue | Perception of substance abuse as a criminal act |
|  | (Waterhouse, 2020) | |
|  | HR = legitimate treatment objective | Recovery = total abstinence |
|  | (Mancini et al., 2008) | (Clark & Wyllie, 2014) |
|  |  | Use during pregnancy perceived as problematic |
|  |  | (Benoit et al., 2014) |
| Status | University students or practitioners |  |
|  | (Davis & Lauritsen, 2016; King, 2020) |  |
| Gender | Male | Female |
|  | (Richards et al., 2021) | |
|  | | |
| **Theme 2: Clients’ characteristics** | | |
| Factors related to SUD | SUD severity: moderate | SUD severity: severe |
|  | (Davis & Lauritsen, 2016; Davis & Rosenberg, 2013; Davis et al., 2017; Lauritsen, 2017; Rosenberg & Davis, 2014; Rosenberg & Melville, 2005; Xin et al., 2022) | (Davis & Lauritsen, 2016; Davis & Rosenberg, 2013; Davis et al., 2017; Lauritsen, 2017; Rosenberg & Davis, 2014; Rosenberg & Melville, 2005; Schippers & Nelissen, 2006; Xin et al., 2022) |
|  | Presence of non-use days; Clear arguments in favor of controlled use | Use fulfils an important psychological function and/or use of other illegal substances |
|  | (Schippers & Nelissen, 2006) | |
|  | Substance consumed: cannabis | Number of previous treatments; Polydrug use |
|  | (Davis & Lauritsen, 2016) | (Davis & Rosenberg, 2013) |
|  |  | Duration and frequency of use |
|  |  | (O’Leary et al., 2018) |
| Therapy considerations | HR used as an intermediate treatment goal | HR used as the final treatment goal |
|  | (Davis & Lauritsen, 2016; Davis & Rosenberg, 2013; Davis et al., 2017; Lauritsen, 2017; Rosenberg & Davis, 2014; Rosenberg & Melville, 2005; Xin et al., 2022) | (Davis & Lauritsen, 2016; Davis & Rosenberg, 2013; Davis et al., 2017; Rosenberg & Davis, 2014; Rosenberg & Melville, 2005; Xin et al., 2022). |
|  | High motivation for change; Treatment for the first time; HR is the only intervention that would keep the client in treatment |  |
|  | (Schippers & Nelissen, 2006) |  |
|  | Pre-contemplation phase of change |  |
|  | (Tatar et al., 2021) |  |
|  | Client goals: harm reduction without total abstinence |  |
|  | (Ogborne & Birchmore-Timney, 1998) |  |
| Medical and/or psychiatric status | Comorbidity with a psychiatric disorder | |
|  | (Clark & Wyllie, 2014; Mancini et al., 2008) | (Davis & Rosenberg, 2013; Davis et al., 2017; O’Leary et al., 2018; Sauvageau, 2018) |
|  |  | First psychotic episode |
|  |  | (Oluwoye & Fraser, 2021) |
|  |  | Comorbidity with a medical condition |
|  |  | (Davis & Rosenberg, 2013; O’Leary et al., 2018) |
|  |  | Poor physical health |
|  |  | (O’Leary et al., 2018; Schippers & Nelissen, 2006) |
| Personal characteristics | Pregnancy period | |
|  | (Barbosa-Leiker et al., 2022; Halladay et al., 2018; Long, 2016; Waterhouse, 2020) | (Benoit et al., 2014) |
|  | Young age | |
|  | (Waterhouse, 2020) | (Davis & Rosenberg, 2013) |
|  |  | Advanced age |
|  |  | (Broussard, 2019; Davis & Rosenberg, 2013; Duke et al., 2020) |
|  |  | Being part of the black community |
|  |  | (Eversman, 2014) |
|  |  | Being homeless |
|  |  | (Davis & Rosenberg, 2013) |
| Relational and family characteristics |  | Having to support your family; Be in a relationship |
|  |  | (Davis & Rosenberg, 2013) |
|  |  | General family environment |
|  |  | (Long, 2016) |
| Professional characteristics |  | Being employed |
|  |  | (Davis & Rosenberg, 2013) |
| Psychological characteristics |  | Emotional instability; Criminal background |
|  |  | (Davis & Rosenberg, 2013) |
|  |  | Impulsivity, self-control |
|  |  | (Schippers & Nelissen, 2006) |
|  |  | Poor cognitive and emotional skills |
|  |  | (Long, 2016) |
|  |  | Family history of drug abuse |
|  |  | (Oluwoye & Fraser, 2021) |
| Social characteristics |  | Having a large social network |
|  |  | (Davis & Rosenberg, 2013) |
|  | | |
| **Theme 3: Factors related to HR** | | |
| HR principles | HR focuses on the client’s needs and objectives |  |
|  | (Davis & Lauritsen, 2016; Duke et al., 2020; Kapur, 2016; Leiker, 2021; Long, 2016; Mancini et al., 2008; Rosenberg & Davis, 2014; Suissa & Bélanger, 2001) |  |
|  | HR creates a non-judgmental framework |  |
|  | (Barbosa-Leiker et al., 2022; Halladay et al., 2018; Leiker, 2021; Mancini et al., 2008) |  |
|  | HR focuses on the present |  |
|  | (Kapur, 2016; Leiker, 2021; Suissa & Bélanger, 2001) |  |
|  | HR is flexible |  |
|  | (Clark & Wyllie, 2014; Duke et al., 2020; Soura, 2016) |  |
|  | HR is non-punitive |  |
|  | (Halladay et al., 2018; Leiker, 2021; Long, 2016) |  |
|  | HR is non-stigmatizing |  |
|  | (Barbosa-Leiker et al., 2022; Kapur, 2016; Suissa & Bélanger, 2001) |  |
|  | HR is preventive |  |
|  | (Barbosa-Leiker et al., 2022; Duke et al., 2020; Tatar et al., 2021) |  |
|  | HR is a motivational intervention or prevention model |  |
|  | (Halladay et al., 2018; Leiker, 2021) |  |
|  | HR is opposed to cannabis criminalization |  |
|  | (Eversman, 2014; Suissa & Bélanger, 2001) |  |
|  | HR values clients |  |
|  | (Kapur, 2016; Mancini et al., 2008) |  |
|  | Achieving minimal goals is perceived as a success, and desired behaviors are rewarded |  |
|  | (Sauvageau, 2018; Suissa & Bélanger, 2001) |  |
|  | HR educates young people about substance use through prevention activities |  |
|  | (Duke et al., 2020; Halladay et al., 2018; Soura, 2016; Suissa & Bélanger, 2001) |  |
|  | HR facilitates access to healthcare services |  |
|  | (Kapur, 2016) |  |
|  | HR does not encourage use, is not dangerous, and is a valid and useful approach |  |
|  | (Mancini et al., 2008) |  |
|  | HR is pragmatic |  |
|  | (Duke et al., 2020) |  |
| HR efficacy and/or misconceptions | HR efficacy | Misconceptions related to HR |
|  | Perception of the benefits and effectiveness of HR | Uncertainties about the efficacy and/or dangerousness of HR |
|  | (Clark & Wyllie, 2014; Ellison, 2017; Mancini et al., 2008; Soura, 2016) | (Broussard, 2019; Clark & Wyllie, 2014; Davis & Rosenberg, 2013; Mancini et al., 2008; Oluwoye & Fraser, 2021; Rosenberg & Davis, 2014) |
|  | HR is effective especially when abstinence is unattainable | Misunderstanding HR practices (e.g., lack of knowledge about treatment application) |
|  | (Davis & Lauritsen, 2016; Tatar et al., 2021) | (Broussard, 2019; Clark & Wyllie, 2014; Leiker, 2021; Long, 2016; Mancini et al., 2008; Sauvageau, 2018) |
|  | HR fosters the client’s engagement | Considering that HR conveys the wrong messages |
|  | (Clark & Wyllie, 2014; Duke et al., 2020; Halladay et al., 2018; Kapur, 2016; Long, 2016; Mancini et al., 2008; O’Leary et al., 2018; Soura, 2016) | (Davis & Rosenberg, 2013; Eversman, 2014; Kapur, 2016; Mancini et al., 2008) |
|  | HR fosters young people’s engagement | Considering that HR promotes substance use |
|  | (Soura, 2016) | (Clark & Wyllie, 2014; Kapur, 2016; O’Leary et al., 2018; Sauvageau, 2018) |
|  | HR puts youth in control of their lives | Considering that HR provokes clients who choose to abstain from substances |
|  | (Duke et al., 2020) | (O’Leary et al., 2018) |
|  | HR promotes the therapeutic alliance | Considering that tolerance of use does not help the individual |
|  | (Mancini et al., 2008; O’Leary et al., 2018; Sauvageau, 2018) | (Clark & Wyllie, 2014) |
|  | HR promotes the quality of life | Considering that HR is an ambiguous approach |
|  | (Mancini et al., 2008; Suissa & Bélanger, 2001) | (Mancini et al., 2008) |
|  | HR promotes reflection and safe decision-making |  |
|  | (Duke et al., 2020; Halladay et al., 2018; Kapur, 2016) |  |
|  | HR promotes the sense of responsibility and autonomy |  |
|  | (Duke et al., 2020; Mancini et al., 2008; Suissa & Bélanger, 2001) |  |
|  | HR contributes to the reduction and/or control of the use |  |
|  | (Duke et al., 2020; Ellison, 2017; Rosenberg & Davis, 2014; Tatar et al., 2021) |  |
|  | HR minimizes the symptoms’ severity |  |
|  | (Ellison, 2017; Tatar et al., 2021) |  |
|  | HR reduces guilt and shame |  |
|  | (Leiker, 2021; Long, 2016) |  |
|  | HR reduces harms to the individual |  |
|  | (Kapur, 2016; Tatar et al., 2021) |  |
|  | HR reduces harms to the pregnant woman and the fetus |  |
|  | (Barbosa-Leiker et al., 2022; Benoit et al., 2014; Long, 2016) |  |
|  | HR promotes life satisfaction, a sense of self-efficacy and improves daily functioning and self-confidence |  |
|  | (Tatar et al., 2021) |  |
|  | HR improves physical health, social and mental well-being |  |
|  | (O’Leary et al., 2018) |  |
|  | HR improves coping strategies |  |
|  | (Ellison, 2017) |  |
|  | HR promotes safe use and client’s integration into the social system |  |
|  | (Kapur, 2016) |  |
| External and other factors | Laws that favor HR adoption | The illegality of cannabis among a specific clientele |
|  | (Kapur, 2016; Suissa & Bélanger, 2001; Waterhouse, 2020) | (Davis & Rosenberg, 2013; Long, 2016; Waterhouse, 2020) |
|  | Ineffectiveness of the War on Drugs | Lack of research on HR |
|  | (Eversman, 2014) | (Broussard, 2019; Kapur, 2016; Leiker, 2021) |
|  | Using a “Consequence Analysis” (CA) | Harm Reduction Acceptability Scale |
|  | (Moore & Mattaini, 2014) | |
|  |  | Lack of funding |
|  |  | (Halladay et al., 2018; Kapur, 2016; Leiker, 2021; Long, 2016; Oluwoye & Fraser, 2021) |
|  |  | Lack of research on HR in the case of pregnancy |
|  |  | (Barbosa-Leiker et al., 2022; Benoit et al., 2014) |
|  |  | Ethical dilemmas in the case of pregnancy |
|  |  | (Benoit et al., 2014; Long, 2016) |
|  |  | HR is not accepted by young individual’s parents |
|  |  | (Kapur, 2016) |
|  |  | Reduced care capacity |
|  |  | (Benoit et al., 2014; Kapur, 2016) |
|  | | |
| **Theme 4: Factors related to the workplace** | | |
| General factors | Management leadership and support | Workplace philosophies that run counter to HR |
|  | (Kapur, 2016; Lauritsen, 2017; Sauvageau, 2018; Soura, 2016) | (Davis & Rosenberg, 2013; Duke et al., 2020; Mancini et al., 2008; Sauvageau, 2018) |
|  | In the case of stakeholders undergoing HR training:   - Presence of a facilitator for HR activities - Facilitator qualities: ability to listen, communicate, negotiate, empathize and be patient | Lack of cooperation and collaboration within the team |
|  | (Soura, 2016) | (Kapur, 2016; O’Leary et al., 2018; Sauvageau, 2018; Soura, 2016) |
|  | Presence of multi-systemic, multi-disciplinary and diverse knowledge and skills | Fear of losing funding or accreditation |
|  | (Kapur, 2016) | (Davis & Rosenberg, 2013) |
|  |  | Unclear policies |
|  |  | (O’Leary et al., 2018) |
| Workplace type | Universities | Detoxification residences; Residential rehabilitation services |
|  | (Halladay et al., 2018) | (Davis & Rosenberg, 2013) |
|  | Homeless services | Community-based organizations |
|  | (King, 2020) | (Oluwoye & Fraser, 2021) |
|  | Hospital | |
|  | (Davis & Rosenberg, 2013; O’Leary et al., 2018) | (Vayda, 2016) |
|  | Assessment, referral, and counseling services; Outpatient agency for SUD and/or mental health disorders | School |
|  | (Ogborne & Birchmore-Timney, 1998) | (Suissa & Bélanger, 2001) |
|  | Community-based organization | |
|  | (Benoit et al., 2014) | (Clark & Wyllie, 2014) |
|  | Private practice | Agency located near apply HR |
|  | (Davis & Rosenberg, 2013) | (Davis & Rosenberg, 2013) |
|  |  | Criminal justice system |
|  |  | (Duke et al., 2020) |
