# Supplementary material for: Facilitators of and obstacles to practitioners’ adoption of harm reduction in cannabis use: a scoping review
Source: Harm Reduct J. 2024 Oct 1;21:178. doi: 10.1186/s12954-024-01093-9 (PMC11445962; doi:10.1186/s12954-024-01093-9)
Supplement: Supplementary file 4 — Additional file 4 [file 12954_2024_1093_MOESM4_ESM.docx]

**Facilitators of and obstacles to practitioners’ adoption of harm reduction in cannabis use: a scoping review**

Roula Haddad, Christian Dagenais, Jean-Sébastien Fallu, Christophe Huỳnh, Laurence D’Arcy, Aurélie Hot

Correspondence to Roula Haddad; [roula.haddad@umontreal.ca](mailto:roula.haddad@umontreal.ca)

**Additional file 4: Clientele, workplace, and population identified in the selected studies**

| **Populations identified in the selected studies** | **References** |
| --- | --- |
| Mental health professionals (e.g., therapists, psychotherapists, psychologists, clinicians, addiction specialists) | (Davis & Lauritsen, 2016; Davis & Rosenberg, 2013; Davis et al., 2017; Duke et al., 2020; Eversman, 2014; Halladay et al., 2018; Lauritsen, 2017; Leiker, 2021; Mancini et al., 2008; Ogborne & Birchmore-Timney, 1998; Oluwoye & Fraser, 2021; Richards et al., 2021; Rosenberg & Melville, 2005; Tatar et al., 2021; Vayda, 2016; Waterhouse, 2020) |
| Counselors | (Benoit et al., 2014; Clark & Wyllie, 2014; Ellison, 2017; Kapur, 2016; King, 2020; Kyser, 2010; Ogborne & Birchmore-Timney, 1998; Richards et al., 2021; Sauvageau, 2018; Schippers & Nelissen, 2006) |
| Managers | (Clark & Wyllie, 2014; Kapur, 2016; Long, 2016; Ogborne & Birchmore-Timney, 1998; Oluwoye & Fraser, 2021; Rosenberg & Melville, 2005; Sauvageau, 2018; Tatar et al., 2021) |
| Social workers | (Halladay et al., 2018; Housenbold Seiger, 2005; Long, 2016; Richards et al., 2021; Suissa & Bélanger, 2001; Vayda, 2016; Waterhouse, 2020; Xin et al., 2022) |
| Front-line healthcare workers (e.g., nurses) | (Barbosa-Leiker et al., 2022; Halladay et al., 2018; Long, 2016; O’Leary et al., 2018; Oluwoye & Fraser, 2021; Tatar et al., 2021) |
| University students (i.e., practitioners in training) | (Abbott-Chapman et al., 2007; Davis & Lauritsen, 2016; Moore & Mattaini, 2014) |
| Psycho-educators or educators | (Richards et al., 2021) |
| Police officers | (Richards et al., 2021) |
| Health professionals (e.g., doctors, psychiatrists) | (Tatar et al., 2021) |
| School staff (e.g., principals, teachers) | (Soura, 2016) |
| Higher education administrators (e.g., deans, directors) | (Broussard, 2019) |
| **Populations’ clientele** | **References** |
| Adults | (Davis & Lauritsen, 2016; Davis et al., 2017; Kapur, 2016; Leiker, 2021; Ogborne & Birchmore-Timney, 1998; Rosenberg & Melville, 2005; Schippers & Nelissen, 2006; Vayda, 2016; Waterhouse, 2020; Xin et al., 2022) |
| Young adults | (Broussard, 2019; Davis & Lauritsen, 2016; Davis et al., 2017; Ellison, 2017; Halladay et al., 2018; Kapur, 2016; Leiker, 2021; Waterhouse, 2020; Xin et al., 2022) |
| Adolescents | (Davis & Lauritsen, 2016; Davis et al., 2017; Kapur, 2016; Leiker, 2021; Soura, 2016; Xin et al., 2022) |
| Individuals with a mental health disorder | (Clark & Wyllie, 2014; Leiker, 2021; Oluwoye & Fraser, 2021; Sauvageau, 2018) |
| Pregnant women | (Barbosa-Leiker et al., 2022; Benoit et al., 2014; Long, 2016) |
| Young people involved in the criminal justice system | (Duke et al., 2020; Richards et al., 2021) |
| Homeless population | (King, 2020; Mancini et al., 2008) |
| Individuals from communities of color | (Eversman, 2014) |
| Young adults with a first psychotic episode | (Tatar et al., 2021) |
| Poly-substance users | (Leiker, 2021) |
| HIV-positive people | (O’Leary et al., 2018) |
| New parents | (Benoit et al., 2014) |
| In prison or on probation population | (Leiker, 2021) |
| **Populations’ workplace** | **References** |
| Outpatient agencies for SUDs and/or mental health disorders | (Davis & Rosenberg, 2013; Davis et al., 2017; Housenbold Seiger, 2005; Lauritsen, 2017; Leiker, 2021; Ogborne & Birchmore-Timney, 1998; Rosenberg & Davis, 2014; Schippers & Nelissen, 2006; Vayda, 2016; Xin et al., 2022) |
| Inpatient or residential addiction rehabilitation services | (Davis & Rosenberg, 2013; Davis et al., 2017; Lauritsen, 2017; O’Leary et al., 2018; Ogborne & Birchmore-Timney, 1998; Rosenberg & Davis, 2014; Sauvageau, 2018; Vayda, 2016; Xin et al., 2022) |
| Community-based organizations | (Benoit et al., 2014; Clark & Wyllie, 2014; Kapur, 2016; Kyser, 2010; Oluwoye & Fraser, 2021; Suissa & Bélanger, 2001; Vayda, 2016; Waterhouse, 2020) |
| Private practice | (Davis & Rosenberg, 2013; Davis et al., 2017; Kyser, 2010; Lauritsen, 2017; Leiker, 2021; Rosenberg & Davis, 2014; Vayda, 2016; Xin et al., 2022) |
| Universities | (Broussard, 2019; Ellison, 2017; Halladay et al., 2018; Lauritsen, 2017; Vayda, 2016; Xin et al., 2022) |
| Detoxification residences | (Davis et al., 2017; Leiker, 2021; Ogborne & Birchmore-Timney, 1998; Rosenberg & Davis, 2014; Rosenberg & Melville, 2005; Xin et al., 2022) |
| Halfway houses | (Davis & Rosenberg, 2013; Lauritsen, 2017; Rosenberg & Davis, 2014; Xin et al., 2022) |
| Hospitals | (Barbosa-Leiker et al., 2022; Davis et al., 2017; O’Leary et al., 2018; Vayda, 2016) |
| Prison settings | (Davis et al., 2017; Kyser, 2010; Richards et al., 2021; Suissa & Bélanger, 2001) |
| Schools | (Kyser, 2010; Soura, 2016; Suissa & Bélanger, 2001) |
| Inpatient agencies (public and/or private) for SUDs and/or mental health disorders | (Eversman, 2014; Leiker, 2021; Suissa & Bélanger, 2001) |
| Homeless services | (King, 2020; Mancini et al., 2008) |
| Assessment, referral, and counseling services | (Ogborne & Birchmore-Timney, 1998; Rosenberg & Melville, 2005) |
| Criminal justice system | (Duke et al., 2020) |
| Early intervention services for first-episode psychosis | (Tatar et al., 2021) |
| Public agencies | (Suissa & Bélanger, 2001) |
| Agencies for pregnant women with SUDs | (Long, 2016) |
| Police sector | (Kyser, 2010) |
