# Supplementary material for: Facilitators of and obstacles to practitioners’ adoption of harm reduction in cannabis use: a scoping review
Source: Harm Reduct J. 2024 Oct 1;21:178. doi: 10.1186/s12954-024-01093-9 (PMC11445962; doi:10.1186/s12954-024-01093-9)
Supplement: Supplementary file 5 — Additional file 5 [file 12954_2024_1093_MOESM5_ESM.docx]

**Facilitators of and obstacles to practitioners’ adoption of harm reduction in cannabis use: a scoping review**

Roula Haddad, Christian Dagenais, Jean-Sébastien Fallu, Christophe Huỳnh, Laurence D’Arcy, Aurélie Hot

Correspondence to Roula Haddad; [roula.haddad@umontreal.ca](mailto:roula.haddad@umontreal.ca)

**Additional file 5: Harm reduction definitions as per the authors of the included studies**

| **Concepts retrieved from HR definitions** | **References** |
| --- | --- |
| Studies that did not define HR | (Barbosa-Leiker et al., 2022; Broussard, 2019; Ellison, 2017; Halladay et al., 2018; Long, 2016; Oluwoye et Fraser, 2021; Rosenberg et Melville, 2005; Sauvageau, 2018; Schippers et Nelissen, 2006; Suissa et Bélanger, 2001; Tatar et al., 2021; Waterhouse, 2020) |
| **HR conceptualization** |  |
| Other appellation for HR: “non-abstinence model” | (Davis et Lauritsen, 2016; Davis et Rosenberg, 2013) |
| No universal definition | (Mancini et al., 2008) |
| **Principals of HR** |  |
| HR does not primarily focus on substance abstinence | (Clark et Wyllie, 2014; King, 2020; O’Leary et al., 2018; Ogborne et Birchmore-Timney, 1998; Richards et al., 2021) |
| HR originates from the field of public health | (Eversman, 2014; Housenbold Seiger, 2005; Kapur, 2016; Lauritsen, 2017; Mancini et al., 2008) |
| HR includes policies, programs, and practices | (King, 2020; Mancini et al., 2008; Moore et Mattaini, 2014; O’Leary et al., 2018; Ogborne et Birchmore-Timney, 1998) |
| HR broadens the spectrum of intervention goals deemed acceptable | (Kyser, 2010; O’Leary et al., 2018; Richards et al., 2021; Vayda, 2016) |
| HR enables access to health services | (Clark et Wyllie, 2014; Kapur, 2016; Kyser, 2010) |
| HR implements educational and preventive strategies | (Duke et al., 2020; Kapur, 2016; Lauritsen, 2017; Soura, 2016) |
| HR is a pragmatic intervention | (Lauritsen, 2017; O’Leary et al., 2018), |
| HR is alternative to traditional treatments | (Kyser, 2010; Vayda, 2016) |
| HR is based on humanistic values | (Housenbold Seiger, 2005; Kapur, 2016) |
| HR brings together different treatment methods | (Housenbold Seiger, 2005; Kyser, 2010) |
| HR reaches the clients where they are | (Kyser, 2010; Lauritsen, 2017) |
| HR disfavors stigmatization | (Richards et al., 2021; Vayda, 2016) |
| HR perceives substance use as a universal societal behavior | (Eversman, 2014) |
| HR is evidence-based | (O’Leary et al., 2018) |
| HR is a non-humiliating and non-punitive model | (Lauritsen, 2017) |
| HR is often presented as the opposite of the abstinence-based model | (Abbott-Chapman et al., 2007) |
| **Efficacity of HR** |  |
| HR reduces the negative legal, medical, professional, social, economic and/or family harms of the substance | (Benoit et al., 2014; Clark et Wyllie, 2014; Davis et Rosenberg, 2013; Davis et al., 2017; Duke et al., 2020; Housenbold Seiger, 2005; King, 2020; Kyser, 2010; Leiker, 2021; Mancini et al., 2008; Moore et Mattaini, 2014; O’Leary et al., 2018; Ogborne et Birchmore-Timney, 1998; Soura, 2016; Xin et al., 2022) |
| HR enables moderate or controlled substance use (reduced amount and/or less frequent use) | (Davis et Rosenberg, 2013; Davis et al., 2017; Housenbold Seiger, 2005; Kyser, 2010; Lauritsen, 2017; Xin et al., 2022) |
| HR ensures safe, secure, and enjoyable use of the substance | (Benoit et al., 2014; Housenbold Seiger, 2005; Kapur, 2016; King, 2020; Lauritsen, 2017; Rosenberg et Davis, 2014) |
| HR promotes a sense of control or self-efficacy regarding the initiation and/or cessation of use | (Benoit et al., 2014; Davis et al., 2017; Xin et al., 2022) |
| HR prevents problematic substance use | (Duke et al., 2020) |
| HR promotes client’s engagement in treatment | (O’Leary et al., 2018) |
| HR aims to improve clients’ health | (Rosenberg et Davis, 2014) |
| HR promotes quality of life | (Lauritsen, 2017) |
| HR promotes client integration into society | (Abbott-Chapman et al., 2007) |
